# Fire needle plus cupping for acute herpes zoster: study protocol for a randomized controlled trial

**DOI:** 10.1186/s13063-020-04599-2

**Published:** 2020-08-06

**Authors:** Ying Zhang, Zuohui Liang, Shihua Li, Ling Yang, Taipin Guo, Yan Xu, Juanjuan Yang, Qiannan Xu, Qing Zhang, Jian Zhao, Cailian Li, Xiuhong Liu

**Affiliations:** 1 The Six Affiliated Hospital of Kunming Medical University, Yuxi, 653100 China; 2 School of Acupuncture-Moxibustion and Tuina and Rehabilitation, Yunnan University of Chinese Medicine, Kunming, 650500 China; 3 The Third People’s Hospital of Yunnan Province, Kunming, 650011 China

**Keywords:** Fire needle plus cupping, Acupuncture, Acute herpes zoster, Randomized controlled trial, Protocol

## Abstract

**Background:**

Acute herpes zoster (AHZ) is a common skin disease caused by invasion of the varicella zoster virus into the ganglia and skin, and the severe pain is the most complaint, which can seriously disturb the normal life of patients. Fire needle plus cupping is a special acupuncture treatment, which is widely used to treat AHZ for its better analgesic effect in China although it has not been fully verified by rigorous randomized controlled trial (RCT).

**Methods/design:**

To test the effect, a three-arm randomized parallel controlled trial protocol has been designed. A total of 105 AHZ patients suffering pain will be randomly divided into three groups in an equal proportion. The interventions are fire needle plus cupping (FC) in group A, famciclovir plus gabapentin (FG) in group B, and fire needle plus cupping plus famciclovir (FCF) in group C. The length for the trial is set for a week time frame. Precisely speaking, the A group (FC) is to carry out 1 treatment per day for the total of 7 treatment sessions within 1 week period. On the other hand, the B group (FG) will take drugs orally three times a day within the trial 1 week. Meanwhile, with its combination element, the C group (FCF) is due to undertake both treatments and drugs as prescribed for A and B groups within the trial week. As an intra-trial arrangement, analgesic medication will be carefully administered for temporary pain release if the sudden intolerable pain appeared. For the primary outcome, this study is due to apply visual analogue scale to identify pain intensity relief. As the secondary outcomes are concerned, this study is aiming to focus on the issues related to changes in substance P and beta-endorphin concentrations in peripheral plasma, as well as those issues of analgesic needs, side effects, symptoms, and physical signs including pain classification, local itching, burning sensation, fever, local lymphadenopathy, skin lesion area, blisters, herpes clusters, vesicular traits, ulcers, and pimples; all these are taken into account for evaluation. For the final stage, the participants are to be followed up for postherpetic neuralgia.

**Discussion:**

The results of this trial aim to provide sufficient evidence on FC treatment over both FG and FCF treatments. It will then give a credible alternative treatment to cut down acute pain and to cure AHZ infection.

**Trial registration:**

Chinese Clinical Trial Registry ChiCTR1800015372. Registered on 28 March 2018.

## Background

Herpes zoster (HZ) is a skin infection disease caused by reactivation of varicella zoster virus which is latent in the sensory ganglia, in which a typical feature is that it causes herpes along the sensory nerve in the corresponding segment, accompanied by severe neuralgia, and a serious impact on the quality of life of patients [1]. Pain symptom is the most common in early stage of disease and many of them without visible herpes appearance. As such, it is easy to be misdiagnosed for other diseases, for example, misdiagnosis of cervical spondylosis or lumbar disc herniation when pain symptom occurs on the neck and shoulders. Another misdiagnosed case example is associated with heart-related disease if the HZ symptom appeared on the left chest. All these misdiagnosed cases are not rare of ordinary occurrences [1]. HZ can occur at any age, but elderly population seems to be most commonly affected. Epidemiological study shows that the incidence, complications, hospitalization rate, and average cost of HZ in China are climbing rapidly with the age increasing [2]. The cumulative incidence with HZ infection is 22.6/1000 among people aged over 50 years old, and this rate climbs up to 3.34 times more among the age group of over 80 years old [2].

The conventional drug therapies for HZ infection can be seen in two phases. Those in acute phase are mainly antiviral, nutritional nerve, and analgesic [3] types of drugs. The antiviral drugs are mainly oral famciclovir and valacyclovir. The nutritional nerve drug is mainly mecobalamin, and the pain relief drugs are mainly paracetamol, tramadol, dezocine, morphine, and gabapentin. However, upon an individual’s health condition, glucocorticoid drugs may be administered orally or intravenously. As in the sequelae phase, treatment drugs are mainly analgesic and antianxiety, and the oral drugs include tricyclic antidepressants, strong opioids, gabapentin, tramadol, and pregabalin. In addition, the drugs such as methylprednisolone and triamcinolone acetonide can be used as nerve-blocking therapy. All of these abovementioned conventional drug schemes yield potential side effects, and it is especially for patients with renal insufficiency or immune system diseases as for these patients the abovementioned schemes are difficult to implement [3]. Furthermore, the cumulative medical cost is high for such treatments on this disease, and patients are not fully satisfied even with the curative effect. With all these downsides of conventional drug therapies, it is said to believe complementary and alternative medicine such as acupuncture may give better results with less side effects and reduce medical costs to treat HZ infection [3, 4]..

Although HZ infection is not a lifelong disease, patients still suffer deeply with excruciating pain, yet it requires faster, economical, and effective treatments to relieve pain and shorter treatment courses. Acupuncture treatment has a positive therapeutic effect on pathological neuralgia, and a number of analgesic mechanisms have also been identified [5–9]. Fire needle and cupping are both traditional methods in acupuncture treatment, and its history can be rooted in many ancient Chinese literatures. The fire needle treatment is to use the alcohol lamp to burn the tip point of the special needle, and then, the needle will be pierced into and pull out the meridian or acupoint quickly—these two steps can yield both ordinary acupuncture and warming effects. When the needle operation is completed, the third step of cupping therapy is implemented immediately. This combining three-step therapy is widely used in the treatment of acute pain symptoms of HZ infection in Chinese hospitals nationwide; however, the test of efficacy of this treatment has not been performed under rigorous RCTs [10–12].

Evidently, by several decades of current and ancient traditional Chinese medicine (TCM) practitioners as well as literatures of TCM, the treatment of fire needle plus cupping (FC) has a positive effect to promote qi and activate blood circulation, which then in turn could increase the nutrition around the lesion and promotes tissue regeneration, and resulting in natural wound healing. Ancient TCM practitioners also found that the heat provided by fire needles can promote microcirculation in the lesion area through the regulation of cutaneous nerves, and it is particularly beneficial to the absorption of inflammation and metabolites [13]. Furthermore, the high temperature of fire needles can generate an effect that directly kills the microorganisms and achieves anti-inflammatory outcomes [14]. Therefore, this trial is aiming along the same line as ancient TCM practitioners and to further provide the fact results in order to validate the analgesic effect on acute herpes zoster (AHZ) treated by trial group A (FC).

## Methods

### Objective

The main purpose of this trial is to gather evidence for the designed treatment in group A (FC) and investigate the effect of designed treatment on relieving the *acute* pain phase in AHZ. Another objective would also be to analyze the correlation between the concentration of substance P and β-endorphin (®-Ep) in peripheral plasma and changes in pain intensity by FC intervention.

### Study design

**Fig. 1:**
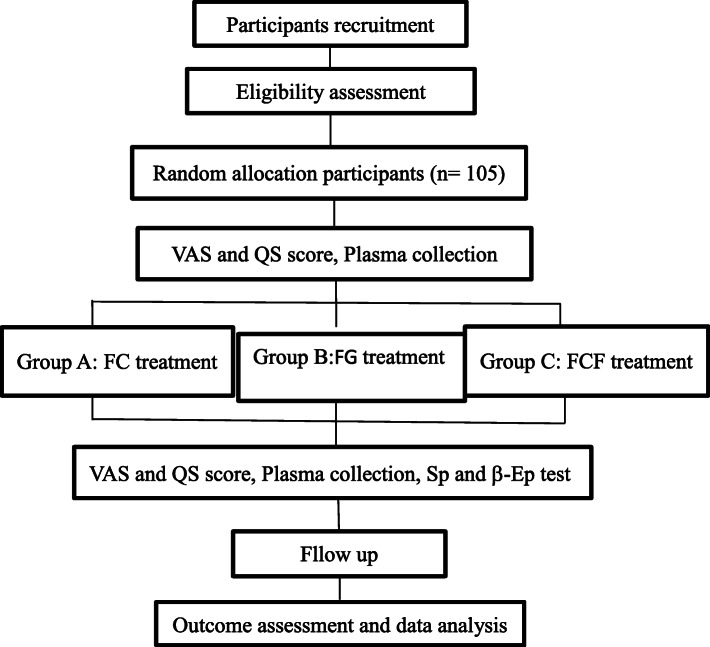
The flow chart of the trial

As shown in Fig. 1, this study is a three-arm open randomized controlled trial consisting of three groups. The interventions are fire needle *plus* cupping (FC) in group A, famciclovir *plus* gabapentin (FG) in group B, and fire needle *plus* cupping *plus* famciclovir (FCF) in group C. After a randomization selection process, group A and group C received a total of 7 treatment sessions of fire needle cupping therapy within 1 week trial time. The administration of gabapentin depends on the needs of the patient of group B. In addition, if the acute pain cannot be endured, patients will be given temporary analgesic medicine.

**Fig. 2:**
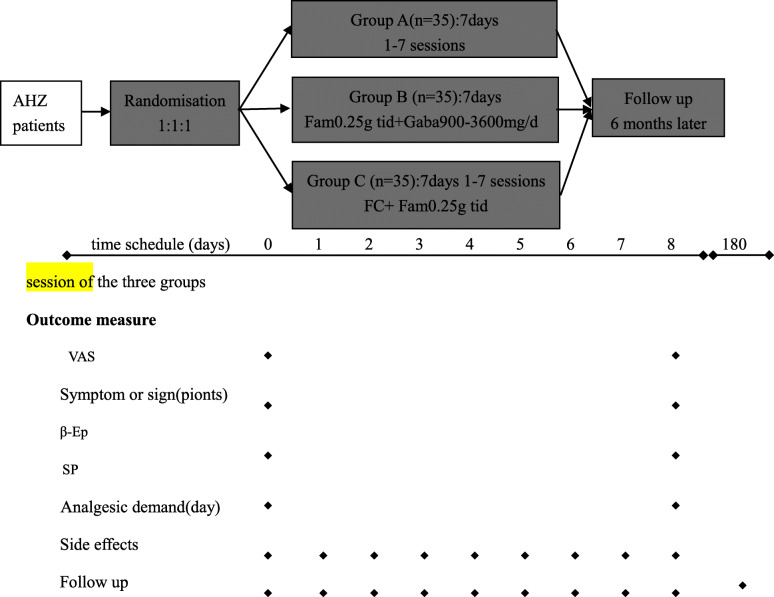
The time schedule of this trial

The visual analogue scale (VAS) scores, symptoms, and physical scores are obtained for pre- and post-treatment periods. The concentrations of SP and β-Ep in peripheral plasma will also be detected, and demand for temporary analgesics and side effects of the patients are recorded daily. As the final stage, after 6 months, the participants are to be followed up for postherpetic neuralgia, 6 months after treatment (Fig. 2).

### Participants and recruitment

This study aims to recruit 105 patients by posting public posters in both local dermatology and acupuncture clinics and the website of the Sixth Affiliated Hospital of Kunming Medical University. When potential participants have read the poster, they can contact the dermatologists Zuohui Liang and Xiuhong Liu through the contact phone number indicated on the poster. Potential patients then got enrolled and signed the consent form if they meet the inclusion criteria by the assessment of dermatologists. Meanwhile, a randomized grouping technique has been conducted. While preparing and unpacking all relevant information, Shihua Li is responsible for such matter. The first formal trial recruitment began in November 2018. The assistant researcher will assess and record the baseline status of the participants (Table 3).

**Fig. 3:**
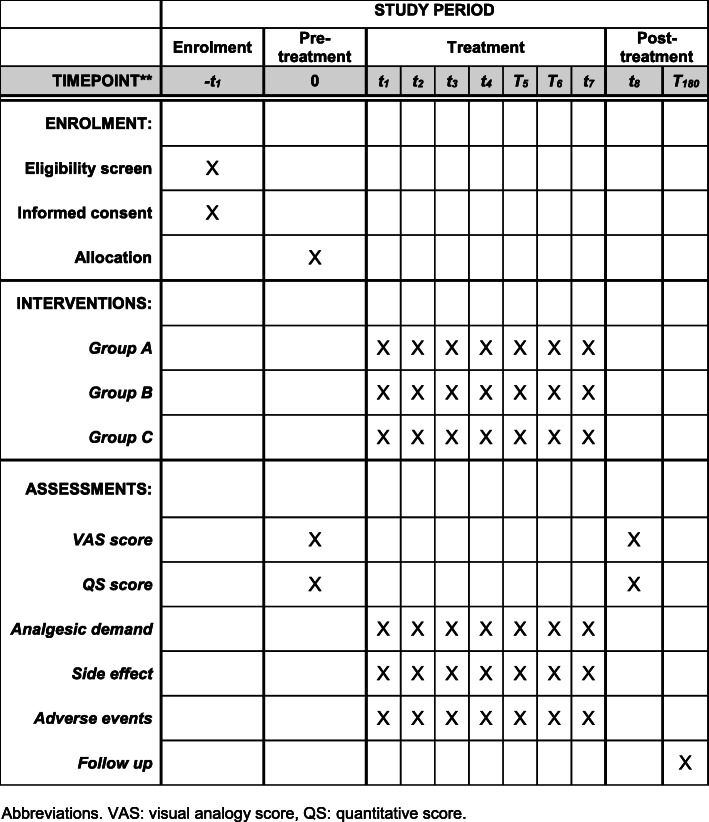
The schedule of enrollment, interventions, and assessments (according to the SPIRIT statement 2013). VAS, visual analogy score; QS, quantitative score

In order to achieve an adequate enrollment rate, we have developed two attractive offerings to gather patients’ attention as well as publicize recruitment notices (Fig. 3). They are as follows: First, all treatment and cost under this trial are free of charge for selected patients *r*. Second, if participants have postherpetic neuralgia 6 months later, they are entitled for another 10 sessions of free comprehensive acupuncture treatments *t*.

### Randomization and allocation concealment

1. Block setting: 105 participants are numbered 1–105 according to the time of participation, the block length is 6, and 16 blocks are set.
2. Obtaining random numbers: start with any two-digit number in the random number table, and take 105 numbers to the right.
3. Grouping: 6 random numbers of each block are sorted from small to large, sorts 1 and 2 are group A, sorts 3 and 4 are group B, and sorts 5 and 6 are group C.
4. Random group concealment: the grouping conditions of participants are packed into 105 envelopes, and all the envelopes are numbered in order and sealed. To ensure the randomization process, the serial number will be printed on the outside of the opaque envelope and the assignment of the group will be sealed on the inside of the envelope.

Shihua Li, an otolaryngologist at the Sixth Affiliated Hospital of Kunming Medical University, is responsible for the above matters, and she does not get involved in the process of treatment and the data collection of this study.

The envelope will be opened according to the patient’s serial number, and the dermatologists will be responsible to inform patients by phone on their random number and assignment.

### Blinding

In order to eliminate operational shortcoming of blinding issues since treatment for FC and FCF group could not be performed blind, we will, instead, first conceal information related to randomized grouping and the results associated with the grouping of subjects, and then, we seek to provide sensory tests (outcome evaluators), data inspectors, and statistical analysts who are not aware of the detailed grouping and treatment of subjects as blind.

### Participating physicians

Participating physicians in the trial are doctors selected from the departments of dermatology and acupuncture and moxibustion at the Sixth Affiliated Hospital of Kunming Medical University. As acupuncturists are responsible for the treatment of FC and FCF, they are all highly qualified and have received a master’s degree in acupuncture and moxibustion as well as have undergone training in unified standardized operation plan.

### Patient and public involvement

Patients and/or public were not involved in the design of this study.

### Participants

#### Inclusion criteria

1. Eighteen to 60 years old, with no gender difference.
2. Skin rash and clustered blister in asymmetrical skin area.
3. Precursor symptoms such as general discomfort and fatigue before rash.
4. Nervous pain in the affected area, skin hypersensitivity.
5. The rash is distributed along the innervated area.
6. Unilateral, not exceeding the midline of the body.
7. Pain intensity as assessed by VAS (0–100 mm) of 50 mm δ pain intensity δ80 mm.

#### Exclusion criteria

1. Insulin-dependent diabetes mellitus or other diseases that affect peripheral sensitivity (e.g., polyneuropathy, chronic pain syndrome)
2. Bleeding tendency (e.g., taking anticoagulants, coagulation dysfunction, thrombocytopenia)
3. Pregnancy or lactation
4. Surgery within the past 3 months
5. Diseases affecting quality of life (e.g., cancer, paralysis)
6. Mental illness (e.g., depression, schizophrenia, dementia) or severe heart/lung/kidney disease
7. Exposure to fire needle, cupping, painkillers, or other complementary and alternative treatments for this disease prior to treatment
8. Contraindications for famciclovir, gabapentin, mecobalamin, paracetamol, tramadol, dextrozine, fire needles, and cupping

### Dropout

#### Case dropout

1. Subjects experienced other comorbidities, complications, or special physiological changes during the trial. They are classified as not suitable to continue the trial.
2. During the trial, serious adverse events and important adverse events occur in the subjects, so they are not suitable to continue the trial, and investigators decide to withdraw.
3. Subjects have poor compliance. Medication compliance is calculated using the tablet counting method. Medication compliance = dose taken/prescription dose × 100%; medication compliance < 80% or missed fire needle plus cupping treatment ≥ 1 time is defined as poor compliance.
4. Violation of the test plan. Subjects change or add drugs other than the study protocol, and received other treatments other than the study protocol during the trial period.
5. The subject withdrew out by himself.
6. Lost to follow-up at the final stage.

#### Management of dropout cases

For dropout cases, researchers should actively take measures to complete the last laboratory test as far as possible in order to analyze its efficacy and safety. For all dropout cases, the test conclusion form and reason for dropout shall be filled in the case report form.

### Intervention

#### Group A

This group will be treated with only FC.

1. Acupoints: the main points are Ashi points (lesion area), corresponding nerve segment Jiaji points, and branch ditch points (SJ6); matching points are selected according to syndrome differentiation, pattern of dampness-heat in the liver meridian with Yang Ling Quan (GB34), pattern of dampness-heat in the spleen meridian with Yin Ling Quan (SP9), and pattern of obstruction of collaterals by blood stasis with blood sea (SP10).
2. Appliances: medium-sized fire needle (diameter 0.4 mm), large-sized fire needle (diameter 0.65 mm), glass fire cup no. 1–5, medical cotton ball, alcohol lamp, lighter, iodophor, etc.
3. Operational methods: routine disinfection of the skin with iodophor with the order of the head, middle, and tail of herpes will be carried out firstly. Holding 95% alcohol lamp by the left hand close to the needle, and to burn the needle in the right hand to whitening by the external flame of the fire, acupuncturist will prick the head of the herpes cluster. Then, the needles will be pricked into the blisters or rashes quickly from the surface of the skin to the base of the herpes. Pricking early-onset herpes at first, for larger pustules or blood blisters (diameter ε0.5 cm) with a large-scale fire, acupuncturist will extrude blister fluid with disinfection cotton ball after puncture, and then cup with a suitable size of glass fire cup for 5–10 min. If the area of the herpes cluster is too large, more than one cup can be used. The remaining acupuncture points are treated with fire needle pricking, and each acupuncture point should be pricked three times. Finally, the skin should be sterilized with iodophor more than one time. The treatment should be performed once a day for a total of 7 days.
4. Skin care: after treatment, the iodophor is used to clean and disinfect the skin and to keep the patient’s skin dry and clean during the treatment.
5. Course of treatment: the course of treatment is 1 week and from one to seven times. If there is no pain any more after one time of FC treatment, the course is just one time. Meanwhile, if the patient is still suffering pain, the FC will continue once a day unless the pain disappears. All the treatments will not be more than seven times no matter the pain or not.

#### Mechanism of fire needle cupping

Based on the theory of traditional Chinese medicine, the main causes of herpes zoster are dampness and heat, which could block the meridians and collaterals, and then led to the stagnation of blood and Qi, and finally, the pain is generated. FC has a strong effect to eliminate the dampness and heat, and make the Qi and blood smooth running, and then, the pain will be alleviated. Previous studies hint that FC can accelerate crusting and shedding of herpes, and also adjust the concentration of substance P in serum [11, 12].

#### Group B

Patients in group B are intervened with famciclovir plus gabapentin (FG). The famciclovir hydrochloride dosage is 0.25 g/time, 3 times a day according to the manufacturer’s (Livzon Pharmaceutical Factory) recommendation. The individual dose of gabapentin is 900–3600 mg per day. According to the manufacturer’s (Jiangsu Hengrui Pharmaceutical Co., Ltd.) recommendation (Table 1), the initial dose of 300 mg per day is gradually increased to 900 mg per day and then increased according to the patient’s needs, and the maximum dose is 3600 mg per day.

**Table 1:** Gabapentin scheme

Day time	8:00 a.m.	14:00 p.m.	22:00 p.m.
1	–	–	300 mg
2	300 mg	–	300 mg
3	300 mg	300 mg	300 mg
Increase depending on patient’s needs			
4	300 mg	300 mg	600 mg
5	600 mg	300 mg	600 mg
6	600 mg	600 mg	600 mg
7 (maximum dose)	1200 mg	1200 mg	1200 mg

Table 1 demonstrates the gabapentin intake scheme used to reach the wanted therapeutic dosage.

#### Group C

In group C, patients will receive the treatment of FC plus famciclovir (FCF). The FC is performed the same as group A, and the usage and dosage of famciclovir hydrochloride are in line with group B.

#### Temporary analgesics

If the patients in the three groups still cannot endure the pain during the treatment or after 1 week treatment, and for all patients who have difficulties to endure pain during the trial, as an intra-trial arrangement, temporarily analgesic medicine will be given to those in need with careful observation. According to the recommendations of the World Health Organization, all three groups are likely to receive standardized analgesic treatment as follows: step 1—non-opioid analgesics (paracetamol 4·1.0 g), 60 mmδ VASδ 50 mm; step 2—moderate-strength opioids (tramadol tablets, maximum dose 600 mg/day), 80 mmδ VASδ 70 mm; step 3—moderate-strength opioids (tramadol injection, 0.1 g, once a day), VAS = 90 mm; and step 4—recommend the use of stronger opioids (dezocine injection, 5 mg, once a day), VAS = 100 mm. Patients are forbad to use other analgesic drugs or therapies. Temporary analgesic demand will be recorded (Table 2).

**Table 2:** Secondary outcomes

**Symptom or sign (points)**	0	1		2		3	
Pain intensity	No	Mild		Medium, tolerable		Severe, unbearable	
Local itching	No	Mild		Medium, tolerable		Severe, unbearable	
Burning sensation	No	Mild		Medium, tolerable		Severe, unbearable	
Rash color	No	Light red		Red, no edema		Red, edema	
No. of blisters	No	1–10		11–15		> 26	
Blister clusters	No	1–2		3–4		4–5	
Ulcer	No	Epidermis		Superficial ulcer		Deep ulcer	
Fever	No	≤ 38 °C		≤ 39 °C		> 39 °C	
Local lymphadenopathy	No	< 0.5 cm		0.5–1 cm		> 1 cm	
Rash area reduction percentage	0	< 30%		< 60%		100%	
**Analgesic demand (day)**	**1**	**2**	**3**	**4**	**5**	**6**	**7**
Paracetamol (g)							
Tramadol (mg)							
Tramadol injection (g)							
Dezocineinjection (mg)							
**Side effects**							

#### Adverse events

Adverse events like symptoms or diseases occurring during the trial will be recorded (Table 2) and assessed at each session of intervention. There may be adverse events of abnormal gastrointestinal reactions, allergic reactions, dizziness, burns, and other medical conditions. The relevance and severity of the adverse events will be assessed. Whether the participant could continue the treatment or not will be decided according to the assessments. Those who suffer harm as a result of the treatment will be compensated in accordance with relevant regulations.

#### Follow-up

To evaluate the incidence of postherpetic neuralgia symptom, a follow-up call is designed and recorded 6 months later posted after the end of the 1-week treatment. (Table 2).

### Outcomes

#### Primary outcome

The primary outcome has been the changes in pain intensity pre- and post-treatment (VAS 0–100 mm, where 0 = *painless* and 100 = *maximum imaginable pain*).

#### Secondary outcomes

First of all, pre- and post-treatment periods, the plasma of the participants is carefully collected and centrifuged and stored in a − 80 °C refrigerator. It is then the substance P and ®-Ep in serum can be detected by an enzyme-linked immunosorbent assay. Quantitative scoring methods have been further applied to evaluate the symptoms and physical signs before and after treatment, including pain intensity, local itching, burning sensation, rash color, numbers of blisters, clusters, ulcers, fever, local lymphadenopathy, and rash area (Table 2).

### Data management and monitoring

The study will be conducted according to the common guidelines for clinical trials (Helsinki Statement, 2008 Chinese Edition, http://www.chictr.org.cn/index.aspx) and will be jointly audited by the Audit Office, Science and Technology Department and Finance Department of Kunming Medical University. Trial auditing will be twice a year since that is the frequency of meeting with the Trial Steering Group of principal investigators. Data will be uploaded to the ResMan Public Management Platform of the China Clinical Trial Registry for adequate quality and safety control. The registration number is ChiCTR1800015372. Therefore, no Data Safety Monitoring Committee is needed. The principal investigator is responsible for project oversight, will make the final decision to terminate the trial, and will have access to the final trial dataset.

### Confidentiality and dissemination

The personal information of all the participants stored on computers is kept on a secure server and will always be kept confidential. All the documentations of this study will be kept in a locked and secure environment (locked office and cabinets) at the Six Affiliated Hospital of Kunming Medical University. The review will be submitted to a peer-reviewed journal prospectively to spread our findings.

### Statistical methods

#### Sample size estimation

We will compare the difference in efficacy of the three groups. Sample size estimation is based on the method of the book of *Health statistics* [15], with type I error alpha = 0.05 and type II error beta = 0.1, using the bilateral test. According to the literature, the cure rates of famciclovir and for fire needle plus cupping for HZ were 37.8% and 76.4%, respectively. It was speculated that the cure rate of famciclovir plus fire needle plus cupping was 80.0%, which was substituted into the formula:

$$ n=\frac{2\lambda }{{\left(2{\sin}^{-1}\sqrt{p_{\mathrm{max}}}-2{\sin}^{-1}\sqrt{p_{\mathrm{min}}}\right)}^2} $$

where *P*_max_ = 0.80 and *P*_min_ = 0.378. The calculated result was a sample size of 32 subjects per group. Therefore, the number of samples required for the three groups was 96. This study required a total of 105 samples adding a 9–10% dropout rate.

#### Statistical analysis

The purpose of this study is to confirm whether the therapeutic effect of experimental therapy (fire needle cupping) is different from that of reference therapy (famciclovir plus gabapentin and fire needle cupping plus famciclovir). SPSS 20.0 statistical software will be used for data analysis. When the main efficacy indicators of individual subjects are missing, the last observation carried forward will be conducted, and the non-main efficacy indicators will not be carried forward. The mean ± standard deviation is used for statistical description of measurement data, and the frequency (constituent ratio) is used for statistical description of counting data. The baseline characteristics will be recorded as in Table 3. The group *t* test (Bonferroni method) will be used to compare the measurement data between groups. All reported *P* values will be two-tailed with 95% confidence intervals. The value of *P* ≤ 0.05 will be considered statistically significant. PPS analysis and Fas analysis will be performed at the same time. SS analysis is used for safety evaluation.

**Table 3:** Baseline characteristics

Characteristics	Value
Age, mean ± SD, year	
Gender, *n* (%)	
Male	
Female	
Onset days, mean ± SD, days	
VAS score, mean ± SD	
Quantitative score, mean ± SD	

Onset days are the time from the patient’s onset of pain or rash to inclusion

Quantitative score is quantitative score of symptoms and signs

## Discussion

This study is a randomized controlled clinical trial with the grouping comparison of FC, FG, and FCF. To our knowledge, so far, this study may be the first clinical trial study that aims to demonstrate the FC effect on AHZ infection with rigorous design and it may also be different from the reference therapy. However, it has gotten our attention that several existing Chinese literatures did report on fire needle and cupping technique to cure for HZ [12–14, 16] but all of them did not take up a rigorous design trial and are lacking of sufficient evidence to prove FC treatment has a positive effect on AHZ.

The key objective of this study is to test the three designed treatment groups on the analgesic effect in acute phase of AHZ disease. We will also pay great attention to the observation of the scores of skin lesions, since skin rash is also the deep concern of patients. We also want to detect variation in serum due to the fact that the substance P and ®-Ep are often regarded as the analgesic mechanism. In addition, the occurrence of postherpetic neuralgia may need to be carefully observed and examined due to its common occurrence and hard to cure nature in AHZ infection. The symptom of acute pain is what the AHZ patients want to solve mostly and cannot approve to participate in an invalid treatment group.

This study is intended to design a rigorous trial protocol that will yield evidence on effective acupuncture technique. In that, this study should be closer enough to the clinical real-world research. Nevertheless, there are confounding factors associated with this designed trial that cannot be completely avoided. First, there is no separate set of sham acupuncture as a control group for FC treatment. Since the first group of FC treatment is already a combination of two types of acupuncture techniques, it is hard to implement another set of sham acupuncture. Another issue of the ethical principle will also make it difficult to perform sham acupuncture for FC treatment since conflicts of interests may happen for patients who have already suffered from severe pain and will not be likely to accept sham acupuncture. Note that, however, the psychological effects cannot be ruled out for lack of sham acupuncture group [17–19]. Second, the blind method is also impossible to perform under the current designed trial since it is not possible for acupuncturists and subjects to do so under the operation of FC treatment. Instead of blinding, the FG and FCF treatments have both been designed to set as the control interventions. As such, the results from FG would be good enough to give evidence of comparison and FCF is also a good way to show whether the combination of drug with FC treatment will yield a better result than the FC treatment alone. Third, as in our clinical practice, acute pain associated with AHZ infection tends to be amplified alongside with the development of the AHZ disease. Therefore, as an intra-trial arrangement, the temporary short-acting painkillers will be permitted to all the selected patients regardless of their group divisions. Having such intra-trial, temporary, and low dose pain release drugs will have no obvious effect on the results. Another limitation of this trial is the psychological factors that cannot be eliminated according to the present design, and the difference in immunity is relative to the recovery of HZ which may lead to differences in prognosis. Finally, according to the actual clinical situation, the drug dose, as well as the number of FC treatment interventions, has certain volatility and does not restrict sternly.

In all, this protocol will act to provide a reference of clinical methodology and may give definite evidence on the effect of FC intervention for AHZ as it is designed.

### Trial status

This trial protocol is version 2.1, dated 24 April 2019. The trial recruitment will be on 10 October 2019, and recruitment will be completed about on 10 October 2020.

## Supplementary information

**Additional file 1.** Ethical Approval Document.

**Additional file 2.** Funding Documentation1.

**Additional file 3.** Funding Documentation2.

**Additional file 4.** Informed consent.

## Data Availability

The full protocol will be available from the corresponding author after identification. Datasets generated or analyzed during the study will not be made public until they are published in a peer-reviewed international journal.

## Abbreviations

HZ: Herpes zoster
AHZ: Acute herpes zoster
RCT: Randomized controlled trial
®-Ep: β-Endorphin
VAS: Visual analogue scale
FC: Fire needle + cupping
FG: Famciclovir + gabapentin
FCF: Fire needle + cupping + famciclovir
TCM: Traditional Chinese medicine
