# Supplementary material for: Fire needle plus cupping for acute herpes zoster: study protocol for a randomized controlled trial
Source: Trials. 2020 Aug 6;21:701. doi: 10.1186/s13063-020-04599-2 (PMC7409425; doi:10.1186/s13063-020-04599-2)
Supplement: Supplementary file 1 — Additional file 1. Ethical Approval Document. [file 13063_2020_4599_MOESM1_ESM.pdf]

**Medical Ethics Committee of Yuxi people's Hospital**

---

**Project name**: **Efficacy observation and mechanism study on the treatment of acute herpes zoster with Zanci of fire needle.**

Project leader: Zhang Ying Professional title: Attending physician

Research institution: The Sixth Affiliated Hospital of Kunming Medical University

Person in charge: Zhang Ying

Address: No. 21, Nieer Road, Hongta District, Yuxi Post code:653100

Project contact: Wang Shuhua TEL: 13908775909 Fax: 0871-65922936

E-mail: lulumager@aliyun.com

Cooperative research unit: The First Affiliated Hospital of Guangzhou University of Traditional Chinese Medicine

Person in charge: Lin Guohua

TEL: Fax: Post code: 510405

Researcher: Lin Guohua Professional title: Chief physician

Researcher: Zhao Lanfeng Professional title: Physicians

Researcher: Lin Ming Professional title: Senior technician

Researcher: Liu Xiuhong Professional title: Attending physician

Researcher: Zhang Bo Professional title: Chief physician

Researcher: Duan Yunqing Professional title: Associate chief physician

Proposed research date: August 1, 2017 - July 31, 2020

Source of research: Government

Type of funder: Government

**Funder name**: Yunnan Provincial Department of Science and Technology (Kunming Medical University)

Funder contact: Wang Zhenyu TEL: 13354657281

Submission of audit information: Experimental program

(Including: Safety data of test supplies, Qualification certificate of production enterprise, Qualification certificate of test supplies provider)

**Abstract**: Taking the patients with acute herpes zoster as the research object and the western medicine as control group. According to the "guidelines for the treatment of herpes zoster in China" issued by the dermatovenerology branch of the Chinese medical association, the patients were treated with fanciclovir hydrochloride, 0.25g, 3 times/day for 7 days. In the treatment group of zanci, the herpes and the corresponding segment jiaji points were treated with zanci of the fire needling. The first 3 days were treated with zanci the fire needling once a day. Since then, the treatment to go once every other day. The course of treatment is 7 days. In the western medicine and zanci group, fanciclovir and zanci were given. The three groups were compared for skin lesions and pain before treatment on the first day, on the fourth and seventh days of treatment (quantitative scoring method was used to evaluate herpes, and visual analogue scale was used to evaluate the efficacy of pain), and detecting substance P and endorphin in peripheral blood of patients before and after treatment. At the end of the course of treatment, patients were followed up for the occurrence of residual neuralgia on the 22rd day, so as to observe the clinical efficacy of the fire needle with zanci in the treatment of acute herpes zoster, and the analgesic mechanism was initially explored.

**Confidentiality points**: The medical records and research materials of the subjects are confidential. When the research results are published, the information related to the subjects will not be disclosed.

**Main researcher qualification evaluation**: The project leader is qualified to conduct the research

**Research program evaluation**: The research project is in line with medical ethics

**Informed consent form**: Informed consent is in line with medical ethics

**Conclusion**: Agreement
No.: 20170730-01

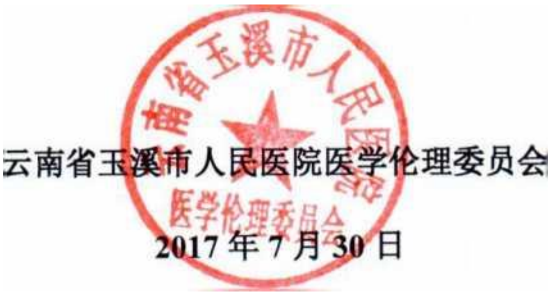


**Medical Ethics Committee of Yuxi people’s Hospital**
