# Supplementary material for: Fire needle plus cupping for acute herpes zoster: study protocol for a randomized controlled trial
Source: Trials. 2020 Aug 6;21:701. doi: 10.1186/s13063-020-04599-2 (PMC7409425; doi:10.1186/s13063-020-04599-2)
Supplement: Supplementary file 2 — Additional file 2. Funding Documentation1. [file 13063_2020_4599_MOESM2_ESM.pdf]

云南省科学技术厅-昆明医科大学应用基础研究联合专项资金 2017 年推荐立项项目公示
Yunnan provincial department of science and technology - kunming medical university applied fundamental research joint special fund recommended for approval in 2017

2017 年云南省科学技术厅昆明医科大学应用基础研究联合专项资金推荐拟立项项目名单
Recommend the list of proposed projects of Yunnan provincial department of science and technology - kunming medical university applied basic research joint special fund in2017

序号<br>serial number	项目名称<br>project name	申报人<br>declarant	所在单位<br>Relying on universities	资助经费（万元）<br>Funding **amount**（RMB：ten thousand ）
……	……	……	……	……
2	火针赞刺法治疗急性期带状疱疹的疗效观察及机制研究<br>responsible unit: Efficacy observation and mechanism study on the treatment of acute herpes zoster with Zanci of fire needle.	郭太品<br>Taipin Guo	昆明医科大学第六附属医院<br>the sixth affiliated hospital of kunming medical university	10
……	……	……	……	……
