# Supplementary material for: Fire needle plus cupping for acute herpes zoster: study protocol for a randomized controlled trial
Source: Trials. 2020 Aug 6;21:701. doi: 10.1186/s13063-020-04599-2 (PMC7409425; doi:10.1186/s13063-020-04599-2)
Supplement: Supplementary file 3 — Additional file 3. Funding Documentation2. [file 13063_2020_4599_MOESM3_ESM.pdf]

云南省科学技术厅-云南中医学院应用基础研究联合专项资金 2017 年推荐立项项目公示
Yunnan provincial department of science and technology - Yunnan university of traditional Chinese medicine applied fundamental research joint special fund recommended for approval in 2017

2017 年云南省科学技术厅-云南中医学院应用基础研究联合专项资金推荐拟立项项目名单
Recommend the list of proposed projects of Yunnan provincial department of science and technology - Yunnan university of traditional Chinese applied basic research joint special fund in2017

序号 serial number	项目名称 project name	申报人 declarant	所在单位 Relying on universities	资助经费（万元）Funding **amount** （RMB：ten thousand ）
……	……	……	……	……
2	基于多模态技术的俞募埋线对胃热-脾虚型腹型肥胖的中枢网络双向调节机制研究 Two-way regulation central mechanism network of shu-muacupoint embedding line for gastro-spleen deficiency type abdominal obesity based on multimodal technology	郭太品 Taipin Guo	云南中医学院 Yunnan university of traditional Chinese medicine	10
……	……	……	……	……
