# Supplementary material for: Fire needle plus cupping for acute herpes zoster: study protocol for a randomized controlled trial
Source: Trials. 2020 Aug 6;21:701. doi: 10.1186/s13063-020-04599-2 (PMC7409425; doi:10.1186/s13063-020-04599-2)
Supplement: Supplementary file 4 — Additional file 4. Informed consent. [file 13063_2020_4599_MOESM4_ESM.pdf]

Informed Consent

Dear patient:

We will invite you to participate in the clinical research of "the Observation of Curative Effect and Mechanism Research on the Treatment of Acute Herpes Zoster by Fire Needle Cupping". Before you decide whether to participate in the study, please read the following carefully as much as possible. It can help you understand the details of the study. If there is still something unclear, please consult your doctor, who will answer your questions.

1. The fire needle cupping method combines the advantages and features of traditional acupuncture methods, such as fire needle therapy, blood pricking therapy, cupping therapy and Zan needling. It is a traditional Chinese medicine treatment of herpes zoster.

2. This study is a randomized clinical control study. You may be divided into a control group or a treatment group. The treatment group is treated with fire needle cupping at the site of herpes and the corresponding segment Jiaji point for 1-7 times. The control group was treated with famciclovir hydrochloride plus gabapperidin or fire needle cupping plus famciclovir hydrochloride (famciclovir 3×0.25g/d P.O., individual dose of 900-3600mg / d. P.O);

3. In case of discomfort to you during the treatment, such as scald, you can report to us at any time and get timely and reasonable treatment; in case of damage related to clinical research, the subject can get appropriate compensation.

4. During the study, you will receive a series of tests, and the content and specific steps of each test will be explained and guided by your doctor. The examination items are as follows: blood routine, urine routine, liver and kidney function, electrocardiogram, chest X-ray examination. Before and after treatment, you will have the determination of substance P and β - endorphin. All of the above tests are safe and will not have adverse effects on your health and condition.

5. Your participation is entirely voluntary. If you are not willing to participate, your treatment will not be affected in any way. At any stage of the clinical research, you have the right to withdraw from the clinical research at any time without any discrimination or retaliation, and your medical treatment and rights will not be affected.

6. If you agree to participate in the study, we sincerely hope that you can cooperate with us to complete the study without special reasons. However, in the course of the study, whether you agree or not, the doctor has the right to suspend you from participating in the study at any time for your benefit.

I have read the above contents in detail or others have read them to me. I have understood the purpose, process and duration of this study, possible benefits, possible risks and inconveniences. Through my independent and full consideration and detailed explanation of the doctor, all questions have been satisfactorily answered. After fully understanding all the contents of the instructions and the advantages and disadvantages of participating in the study, I will voluntarily participate in the study. I will comply with the requirements of the study plan, and I am willing to solve any problems through friendly consultation with researchers, and seriously complete the study.

Signature of patient: Signature of family: (relationship) Date:

Signature of doctor: Date:

知情同意书 Informed Consent

尊敬的患者:

您的医生已经决定对您采用化学药物治疗。我们将邀请您参加“火针赞刺法治疗带状疱疹的疗效观察及机制研究” 的临床研究。在您决定是否参加这项研究之前，请尽可能仔细阅读以下内容，它可以帮助您了解该项研究的详细信息，如仍有不清楚之处，请咨询您的医生，他或她将解答您的疑问。

1、火针赞刺法汇集了火针疗法、刺血疗法、火罐疗法与赞刺针法等传统针灸方法的优势特色，是治疗带状疱疹的中医特色疗法。

2、本研究为随机临床对照研究，您有可能被分到对照组或治疗组，治疗组于疱疹部位及相应节段夹脊穴行火针赞刺法治疗 1-7 次。对照组予口服盐酸泛昔洛韦加加巴喷丁治疗或火针赞此法加口服盐酸泛昔洛韦治疗（盐酸泛昔洛韦 3×0.25g/d p.o.，个体化剂量的加巴喷丁 900-3600mg / dp. p o.）；（c）火针加拔罐加泛昔洛韦静滴阿昔洛韦 5mg/kg，每 8 小时一次，口服泼尼松 20mg/d，外擦阿昔洛韦软膏及激光照射治疗。

3、如在治疗过程中给您带来不适，例如烫伤，您可随时向我们反映并得到及时、合理的处理；若发生与临床研究有关的损害，受试者可得到适当的补偿。

4、研究期间您将接受一系列检查，每次检查的内容和具体步骤，您的医生将负责给予说明和指导。检查项目如下: 血常规、尿常规、肝肾功能、心电图、胸部 X 光检查，治疗前和治疗后您将进行 P 物质、β-内啡肽的测定。以上所有检查均很安全，不会对您的健康和病情产生不良影响。

5、您的参与完全出于自愿，如您不愿意参加，不会再任何方面影响医生对您的治疗；在临床研究的任何阶段，您均有权随时退出临床研究而不会遭受到任何歧视或报复，您的医疗待遇与权益不受影响。

6、如果您同意参加本项研究，在没有特殊原因的情况下，我们真诚地希望您能与我们合作完成该项研究。但在研究过程中，无论您是否同意，医生有权从您的利益出发在任何时候中止您继续参加此研究。

我已详细阅读过或其他人已经向我读过以上内容，已经了解本研究的目的、过程和期限、可能的受益和可能发生的风险和不便，通过我独立、充分的考虑和医生的详细解释，所有疑问已得到圆满解答。在充分了解须知全部内容及参加研究带来的利弊后我自愿参加这项研究，我将遵守本研究方案要求，愿意与研究者友好协商解决任何问题，认真完成本研究。

患者签字: 家属签字: (关系_) 日期: 年 月 日

医生签字: 日期: 年 月 日
